# Editorial: Integrating molecular research into clinical psychiatry: An application to schizophrenia and mood disorders

**DOI:** 10.3389/fpsyt.2023.1085663

**Published:** 2023-03-10

**Authors:** Tetsufumi Kanazawa, Shuken Boku, Hiroyuki Toda, Ryouhei Ishii

**Affiliations:** ^1^Department of Neuropsychiatry, Osaka Medical and Pharmaceutical University, Osaka, Japan; ^2^Department of Neuropsychiatry, Faculty of Life Sciences, Kumamoto University, Kumamoto, Japan; ^3^Department of Psychiatry, School of Medicine, National Defense Medical College, Saitama, Japan; ^4^Department of Occupational Therapy, Graduate School of Rehabilitation Science, Osaka Metropolitan University, Osaka, Japan; ^5^Department of Psychiatry, Graduate School of Medicine, Osaka University, Osaka, Japan

**Keywords:** schizophrenia, mood disorder, basic research, practice, implementation

In recent years, significant progress has been made in brain science research applied to molecular biology, and research results have provided insights into the pathophysiology of psychiatric disorders. Additionally, genetic research, especially comprehensive analysis of big sample sizes, has yielded results with unprecedented scientific reliability. Furthermore, various types of brain imaging analysis methods are expected to contribute to the diagnosis of psychiatric disorders, such as schizophrenia (SCH) and bipolar disorder (BD), because of the advances in functional methodology as well as structural retrieval. On the other hand, conventional descriptive methodologies are still the mainstream in actual clinical practice in psychiatry, and research results that spill over into general clinical practice must be said to be limited.

The purpose of this Research Topic was to provide a comprehensive picture of the clinical application of the latest research findings to SCH, BD, and other psychiatric disorders, and to provide an opportunity to put basic research into actual psychiatric practice. A total of seven manuscripts (five original papers, one mini-review, and one opinion piece) were accepted for this topic, all of which contain important findings and new research methods (postmortem expression profiling, transcriptome analysis of peripheral blood and brain tissue, EEG microstates, etc.). The goal of each researcher is to elucidate the pathophysiology of psychiatric disorders using basic research methods and to apply the results to the treatment of real patients.

In recent years, it has been suggested that molecular changes in the central nervous system (CNS) play a key role in the pathogenesis of SCH. Transcriptomics analysis, which contributes to the elucidation of changes in upstream regulatory sequences, is increasingly being used to understand the pathogenesis of SCH. However, the application of transcriptomics analysis in the CNS has been limited by the difficulty of collecting postmortem brain tissue. On the other hand, peripheral blood mononuclear cells (PBMCs) are an important target for SCH research because they can be easily collected from patients and partially reflect molecular changes in the CNS. To search for biological. pathways showing similar expression patterns in both brain tissue and PBMCs, Song et al. used a transcriptome dataset of brain tissue from SCH patients downloaded from public databases. They also used transcriptomic techniques to elucidate mRNA expression in PBMCs from 10 drug-naive SCH patients and 20 normal controls (NC). Biological pathways with similar expression patterns in brain tissues and PBMCs were then analyzed by differential expression analysis, weighted gene co-expression network analysis (WGCNA), and pathway analysis. They validated differential expression gene (DEG) expression levels by real-time fluorescence quantitative polymerase chain reaction (qPCR) and found that pathways related to phospholipid metabolism, ribosomal signaling, and energy metabolism showed similar expression patterns in brain tissues and PBMC from SCH patients. They identified 542 DEGs, 51 DEGs, 732 DEGs, and 104 DEGs in PBMC, dorsolateral prefrontal cortex, anterior cingulate gyrus, and nucleus accumbens, respectively. Pathway analysis revealed that these DEG clusters were enriched in several biological pathways mainly related to phospholipid metabolism, ribosomal signaling, and mitochondrial oxidative phosphorylation. Their groundbreaking results provided further evidence of dysfunction in phospholipid metabolism, ribosomal signaling, and energy metabolism in patients with SCH, which give us new insights into the pathogenesis of SCH and a new approach to explore potential biological markers of peripheral blood in SCH

Despite the fact that the atypical antipsychotic clozapine is an established treatment for treatment-resistant SCH, the molecular mechanism of clozapine remains poorly understood because of the varying genetic backgrounds of patients. Kikuchi et al. sought to overcome this challenge by focusing on case combinations of monozygotic twins with treatment-resistant SCH in which one twin responded well to clozapine treatment while the other did not. They generated neurons from induced pluripotent stem (iPS) cells obtained from these patients, compared the transcriptome profiles of the mock and clozapine-treated neurons, and applied genome-wide DNA methylation profiling to investigate the mechanisms behind the changes in gene expression. First, based on statistical analysis, sites with different degrees of methylation were extracted from each twin. Next, they combined this DNA methylation profiling with transcriptome profiling using previously obtained RNA-seq data. They found that among the genes with altered methylation and expression, the proportion of genes associated with neuronal and synaptic function differed between clozapine responders and non-responders (35.7 and 6.7%, respectively). This trend was observed even excluding the underlying differences between responders and non-responders. These results suggest that the effective action of clozapine may correct neuronal and synaptic dysfunction in SCH *via* changes in methylation.

Insulin-like growth factor-1 (IGF-1) is a tropic mediator associated with neuronal proliferation, development, and growth. Although IGF-1 may be associated with the pathophysiology of SCH, this association has been still controversial. To clarify the association between the pathophysiology of SCH and IGF-1, Okamoto et al. investigated the relationship between serum IGF-1 levels and psychiatric symptoms in 20 age- and sex-matched NC and 65 patients with chronic SCH. Although they found no significant differences in serum IGF-1 levels between SCH and NC, but serum IGF-1 levels had a significant negative correlation with PANSS total score general score, they found a significant correlation between serum IGF-1 levels and age. They concluded that serum IGF-1 levels do not distinguish NC from SCH and suggested that the association between psychiatric symptoms and serum IGF-1 levels may be complicated in patients with chronic SCH.

Recent studies have indicated that inflammation may be related to the pathogenesis of SCH. Previous studies measuring inflammatory mediators have found elevated levels of inflammatory cytokines in the brain and blood of SCH patients. Several postmortem brain studies have also reported altered expression of inflammatory cytokines. However, it was not clear how these elevated inflammatory cytokines interact with other inflammatory mediators and relate to the pathogenesis of SCH. The superior temporal gyrus (STG) is a brain region suspected to be associated with hallucinations and thought disorder in the pathogenesis of SCH. Using brain tissue from several postmortem brain banks, Izumi et al. examined the expression of 30 inflammatory mediators in the STG of 24 SCH patients and 26 controls using multiplexing analysis. The results showed that there was little difference in the expression of inflammatory mediators in STG between the subject and SCH groups, but the expression of interleukin (IL)-1-α and interferon (IFN) gamma-induced protein (IP)-10 was decreased, and IFN-α was increased in the SCH group, although not significant after multiple testing. Furthermore, clustering based on the expression patterns of inflammatory mediators and pathway analysis of upstream transcription factors depicted that the suppression of IL-1α and IP-10 protein expression may be induced by the regulation of a common upstream pathway. Their comprehensive protein analysis of STG revealed previously unreported protein alterations, and these results provide insight into post-inflammatory changes in chronic SCH.

EEG microstates are defined as transient, patterned, metastable states of EEG. These patterns tend to last from milliseconds to seconds and have been interpreted as the most basic instances of human cognitive neurological processing. Wang et al. evaluated the utility of EEG microstates as a biomarker to distinguish patients with BD from those with SCH. They analyzed EEG data from 20 SCH patients, 26 BD patients, and 35 NCs, measured in a resting state with eyes closed. EEG microstate analysis resulted in a global clustering of four microstates (A-D) among SCH patients, BD patients, and HC. The results showed a greater presence of microstate B in BD patients, a smaller presence of microstate classes A and B, a greater presence of microstate class C, and a smaller presence of D in SCH patients. They also found a higher frequency of switching between microstates A and B and between microstates B and A in BD patients compared to SCH patients and HC, and a lower frequency of switching between microstates C and D and between microstates D and C in BD patients compared to SCH patients. They found abnormal features of microstates A and B in BD patients and abnormal features of microstates A, B, C, and D in SCH patients. These features may indicate potential abnormalities in SCH and BD patients that affect the allocation of neural resources and appropriate transitions between different active states.

The coronavirus pandemic had a major impact on people's emotions, including fear of infection, anxiety, and depressed mood. In terms of preventing the spread of the virus, it would be ideal for all people to avoid interpersonal contact in all situations. Ikeda et al. raise the issue of the need to establish a telemedicine system for the treatment of mental illness in order to solve this problem. Currently, online diagnosis through Digital Transformation (DX) technology, as typified by telemedicine, is becoming increasingly popular; DX technology can realize an efficient mental health care system by consolidating necessary information into a smart device and minimizing the burden on medical professionals and patients Potential. Transcranial direct current stimulation (tDCS) is a NIBS technique that uses a simple device that delivers a weak DC current between electrodes placed on the scalp. tDCS's high safety profile has led to rapid progress in high-quality research on tDCS regarding cognition and emotion regulation, and treatment with tDCS devices is considered suitable for telemedicine Therapy with tDCS devices is considered to be suitable for telemedicine. It is ideal for home care because the treatment is entirely delivered by electronic devices and changes in stimulation parameters can be easily managed. Therefore, several ideas for telemedicine using tDCS have been proposed. They discussed the future of DX-enabled tDCS-telemedicine systems in light of the need for telemedicine options highlighted by the COVID-19 epidemic; DX technology has the potential to centralize necessary information on smart devices and minimize the burden on healthcare professionals and patients, thereby creating an efficient They discussed the future of DX-compatible tDCS-telemedicine systems in light of the need for telemedicine options highlighted by the COVID-19 epidemic. They proposed that improved technology integrating tES and neurophysiology is needed to ensure safety and efficacy. They also emphasize that in order to utilize tES in telemedicine, it is essential to establish a system in which medical institutions are comprehensively evaluated and consulted and followed up by experts. This concept they propose cannot be realized by the medical industry alone, but requires collaboration with industry and social systems. Now that the productivity and importance of telemedicine has been recognized by the coronavirus pandemic, this perspective is realistic and is the future vision we should strive for.

Depression has a variety of etiologies, and factors have been identified that explain individual differences in pathophysiology and treatment efficacy, including neural circuits, biotypes, biopsychosocial markers, genetics, and metabolomics. While precision approaches promise to enhance diagnostic and treatment decisions, there are significant challenges that impede their clinical application. These include the clinical diversity of psychiatric disorders, the technical complexity and cost of multi-omics data, the need for specialized training in precision medicine for medical staff, and ethical concerns such as protecting the privacy and security of patient data and maintaining health equity Deif and Salama reviewed the literature to review recent findings in the conceptualization and treatment of depression from a precision medicine perspective and to discuss potential challenges and future directions in the application of precision psychiatry to the treatment of depression. Computer and information technology has advanced over the past several years, facilitating research using highly accurate and relevant clinical data sets and, in some cases, predicting disease and treatment outcomes from artificial intelligence-based modeling systems. Nevertheless, several challenges may still limit their clinical utility. For example, among the challenges they cite are the fact that the majority of clinical trials recruit mild and moderate patients, with the exception of those with more severe symptoms, and are not representative of the actual patient population; the need for adequate training in various aspects of precision medicine; the health disparities that exist between patients who can afford precision medicine services and those who cannot; and the existence of health disparities and inequities between patients who can afford precision medicine services and those who cannot, difficulties in protecting data security and privacy, and concerns about marginalization in mental health services caused by the use of artificial intelligence in clinical decision making.

In summary, this Research Topic presented a comprehensive picture of the clinical application of the latest research findings to SCH, BD and other psychiatric disorders, and to provide an opportunity to put basic research into actual psychiatric practice. As a result, these papers elucidated the pathophysiology of psychiatric disorders using basic research methods and to apply the results to the treatment of real patients with new evidence and threw impacts on new directions for expanding new possibility of neuroscience for basic and clinical application on clinical practice in psychiatry.

## Author contributions

RI and TK discussed about this Research Topic and wrote the manuscript. SB and HT gave significant advice and helped in editing the manuscript. All authors contributed to the article and approved the submitted version.

